# M1 Macrophage Derived Exosomes Aggravate Experimental Autoimmune Neuritis via Modulating Th1 Response

**DOI:** 10.3389/fimmu.2020.01603

**Published:** 2020-07-23

**Authors:** Tong Du, Chun-Lin Yang, Meng-Ru Ge, Ying Liu, Peng Zhang, Heng Li, Xiao-Li Li, Tao Li, Yu-Dong Liu, Ying-Chun Dou, Bing Yang, Rui-Sheng Duan

**Affiliations:** ^1^Department of Neurology, Shandong Provincial Qianfoshan Hospital, Shandong University, Jinan, China; ^2^Department of Neuronal Electrophysiology, The First Affiliated Hospital of Shandong First Medical University, Jinan, China; ^3^Department of Neurology, The First Affiliated Hospital of Shandong First Medical University, Jinan, China; ^4^College of Basic Medical Sciences, Shandong University of Traditional Chinese Medicine, Jinan, China

**Keywords:** macrophages, exosomes, T helper 1 (Th1) cells, IFN-γ, experimental autoimmune neuritis (EAN), Guillain–Barré syndrome (GBS)

## Abstract

Guillain–Barré syndrome (GBS), an immune-mediated disorder affecting the peripheral nervous system, is the most common and severe acute paralytic neuropathy. GBS remains to be potentially life-threatening and disabling despite the increasing availability of current standard therapeutic regimens. Therefore, more targeted therapeutics are in urgent need. Macrophages have been implicated in both initiation and resolution of experimental autoimmune neuritis (EAN), the animal model of GBS, but the exact mechanisms remain to be elucidated. It has been increasingly appreciated that exosomes, a type of extracellular vesicles (EVs), are of importance for functions of macrophages. Nevertheless, the roles of macrophage derived exosomes in EAN/GBS remain unclear. Here we determined the effects of macrophage derived exosomes on the development of EAN in Lewis rats. M1 macrophage derived exosomes (M1 exosomes) were found to aggravate EAN via boosting Th1 and Th17 response, while M2 macrophage derived exosomes (M2 exosomes) showed potentials to mitigate disease severity via a mechanism bypassing Th1 and Th17 response. Besides, both M1 and M2 exosomes increased germinal center reactions in EAN. Further *in vitro* studies confirmed that M1 exosomes could directly promote IFN-γ production in T cells and M2 exosomes were not capable of inhibiting IFN-γ expression. Thus, our data identify a previously undescribed means that M1 macrophages amplify Th1 response via exosomes and provide novel insights into the crosstalk between macrophages and T cells as well.

## Introduction

Guillain–Barré syndrome (GBS), first described over a century ago, epitomizes immune-mediated disorders against peripheral nervous system (1, 2). Nowadays, it still stands as the leading cause of acute flaccid paralysis worldwide, with an incidence of 0.81–1.89 cases per 100,000 individuals (3). Clinical and electrophysiological manifestations help to discriminate subtypes and variants of GBS, which usually differ in prognosis. Despite the fact that many patients benefit from intravenous immunoglobulins or plasma exchange, GBS is still a life-threatening condition. The overall mortality of GBS varies between regions, reported to be 3–5% in Europe and North America and reaching to 17% in certain area, and some patients suffer from devastating sequelae for months or years (2, 4–6). Large efforts have been made to uncover the pathogenesis of GBS. Experimental autoimmune neuritis (EAN) is a well-accepted animal model mimicking acute inflammatory demyelinating polyneuropathy (AIDP), which represents the major subtype of GBS, and studies from EAN and human pathological data have illuminated the immune rationale underlying AIDP, in which CD4+ T cells and macrophages play dominant roles (2).

Macrophages are the key component of the innate immunity. They maintain high plasticity and exquisitely scan external signals, exhibiting distinct phenotypes and functions in response to environmental changes. M1 macrophages, activated by inflammatory insults and T helper 1 (Th1) cell derived IFN-γ, can in turn shape the magnitude of adaptive immunity, and most studies about this process has so far been focused on cytokines, among which IL-12 signaling has been most studied (7–9). M1 macrophages have well-characterized roles in the progressive stage of AIDP (10–12). Inhibition of glycolysis by 2-DG, which is essential for metabolic reprogramming in M1 macrophages, attenuates both initiation and progression of EAN (13). Recent studies have also suggested the importance of M2 macrophages, which can arise in Th2 settings, in timely termination of EAN and subsequent promotion of tissue repair (7, 14, 15). Considering the implications in both inflammation initiation and resolution, macrophages are emerging as a promising therapeutic target for GBS and other inflammatory disorders. As effective functioning of macrophages requires well-orchestrated communication between macrophages and other types of immune cells, there arise demands for comprehensive explorations of the crosstalk network under certain contexts.

Exosomes, with size ranging from 50 to 150 nm, belong to extracellular vesicles (EVs) and are originated from multivesicular endosomes (MVEs) (16). Exosomes were initially considered as waste carriers of cells, but subsequent insights into their potentials for exchange of cellular components, including nucleic acids, proteins, and lipids, have greatly expanded our knowledge of intercellular communication (17). A growing body of evidence has highlighted the importance of exosomes for macrophage biology under both homeostatic and pathological circumstances. Macrophage derived exosomes are involved in maintaining bone homeostasis (18). In tumor microenvironment, exosomes derived from tumor associated macrophages (TAMs) have been proven to promote migration and invasion of colorectal cancer cells and to regulate aerobic glycolysis of breast cancer cells, via transferring certain miRNAs or lncRNAs, respectively (19, 20). Exosomes also contribute to crosstalk between nicotine-treated macrophages and vascular smooth muscle cells, exacerbating the development of atherosclerosis (21). Furthermore, systematic regulation of insulin sensitivity by miRNA-containing exosomes from adipose tissue macrophages (ATMs) has also been well-documented (22). However, the roles of macrophage derived exosomes in inflammatory disorders and the mechanisms behind have been understudied and remain blank in EAN/GBS.

In the current study, we sought to evaluate the effects of macrophage derived exosomes on the development of EAN and investigated the cellular mechanisms involved, with particular focus on their potential effects on T cell subtypes. We found that M1 macrophage derived exosomes (M1 exosomes) and M2 macrophage derived exosomes (M2 exosomes) differently affected the development of EAN and Th1 response *in vivo*. Further *in vitro* studies showed, for the first time, that M1 macrophage-derived exosomes could modulate IFN-γ expression in T cells in a direct manner. These findings not only suggest that M1 exosomes promote the development of EAN, at least in part, via directly modulating Th1 response, but also delineate a new way of crosstalk between macrophages and T cells.

## Materials and Methods

### Experimental Animals

Lewis rats were purchased from Vital River Laboratory Animal Technology Co. (Beijing, China) and maintained at the specific pathogen-free animal facility of our institute, fed *ad libitum* on a 12/12-h light/dark cycle and monitored periodically for their health status. Female rats (170–190 g) were used in this study. All animal procedures were reviewed and approved by the Institutional Animal Care and Use Committee at Shandong University School of Medicine.

### Reagents

Reagents used in this study are described in the corresponding sections and all antibodies used in flow cytometry and immunoblotting are also listed in Supplementary Table 1 for easy reference.

### Bone Marrow Derived Macrophages (BMDMs) Preparation and Polarization

To obtain BMDMs, bone marrow cells from femurs and tibias were harvested from healthy Lewis rats and passed through a 70 μm cell strainer. Erythrocytes were then removed by a RBC lysis solution (Biolegend, 420301). Remaining cells were resuspended at a concentration of 2 × 10^6^ cells/mL in IMDM (Biological Industries, 01-058-1A) supplemented with 10% FBS (Biological Industries, 04-002-1A), 1% penicillin-streptomycin (Gibco, 15140122) and 10 ng/mL recombinant rat M-CSF (Biolegend, 556902). 10 mL cell suspension was then seeded in 100 mm non-tissue-culture treated dishes for flow cytometry analysis or in tissue-culture treated dishes for any other experiments, and cultured in a humidified incubator with 5% CO_2_ at 37°C. Equal volume of fresh differentiation media were added on day 3 and half of the media was replaced on day 5, providing M-CSF for macrophage differentiation.

On day 7, macrophages were treated with LPS (100 ng/mL; Sigma-Aldrich, L4391) + recombinant rat IFN-γ (20 ng/mL; Biolegend, 598802) for M1 polarization and with recombinant rat IL-4 (20 ng/mL; Biolegend, 776902) for M2 polarization for indicated time duration.

### Macrophage Derived Exosome Isolation

For exosome isolation and other *in vitro* experiments evaluating the effects of exosomes, exosome-depleted FBS was used, which was prepared by ultracentrifugation at 100,000 g overnight. Macrophages were polarized to M1 or M2 phenotypes for 48 h. The conditioned media were collected and centrifuged at 300 g for 10 min, followed by centrifuging at 2,000 g for 10 min. Supernatant was preserved and centrifuged at 10,000 g for 30 min at 4°C to remove large vesicles. The resultant supernatant was centrifuged at 100,000 g for 90 min at 4°C using a Thermo Scientific Sorvall WX 80+ ultracentrifuge and pellets were resuspended in phosphate-buffered saline (PBS) and subjected to another round of ultracentrifugation. Exosome pellets were recovered in PBS and ready for subsequent experiments. Protein concentration in the solution was measured using Pierce BCA Protein Assay Kit (Thermo Scientific, 23227) to indicate the amount of exosomes.

### Characterization of Exosomes

For transmission electron microscopy analysis of exosomes, 20 μL of exosome suspension was transferred onto Formvar/carbon grids and negatively stained with 2% uranium acetate solution for 1 min. Data were acquired by Tecnai G2 Spirit BioTWIN.

Flow cytometry analysis of exosomes was performed as previously described (23), with some modifications. Briefly, 5 μg of exosomes or equal volume of negative control solution were incubated with 1.6 μL of 4 μm-diameter aldehyde/sulfate latex beads (Invitrogen, A37304) in a total volume of 50 μL for 15 min at room temperature, followed by gentle shaking in 1 mL of PBS for 2 h. The reaction was stopped by incubation for 30 min in 100 mM glycine. Exosome-bound beads were washed by 2% BSA twice and centrifuged at 3,000 g for 10 min. These beads were then incubated with allophycocyanin (APC)-conjugated anti-CD81 (Biolegend, 104909) for 30 min at 4°C, followed by washing, and were ready to be examined.

Immunoblotting was performed to detect exosome-enriched proteins. Twenty microliter of isolated exosomes was loaded, and exosomal proteins were separated by SDS-PAGE and thereafter transferred onto polyvinylidene difluoride (PVDF) membranes. Blots were blocked with 5% non-fat dry milk at room temperature for 1 h and probed with primary antibodies against TSG101 (Proteintech, 14497-1-AP) and ALIX (Abcam, ab186429), followed by incubation with corresponding HRP-conjugated secondary antibodies at room temperature for 1 h. The blots were then visualized with chemiluminescent HRP substrate (Millipore, WBKLS0100).

### Establishment of EAN and Assessment of Clinical Scores

Bovine peripheral myelin (BPM) was prepared in accordance with our previous report (24). To induce EAN, rats were immunized at the base of tail subcutaneously with a total of 200 μl inoculum, containing 1 mg dissolved BPM emulsified in an equal volume of incomplete Freund′s adjuvant (Sigma-Aldrich, F5506) containing 0.45 mg *Mycobacterium tuberculosis* (strain H37RA; Difco). Rats were weighted daily and evaluated for neurogenic signs of EAN in accordance with the following scale: 0, normal; 1, reduced tonus of the tail; 2, partial tail paralysis; 3, complete tail paralysis or absent righting reflex; 4, gait ataxia; 5, mild paresis of the hind limbs; 6, moderate paraparesis; 7, severe paraparesis of the hind limbs; 8, tetraparesis; 9, moribund; and 10, death.

### Macrophage Derived Exosome Treatment

EAN rats were randomly assigned to three groups, and intravenously injected with 20 μg of M1 or M2 exosomes, or with an equal volume of vehicle every other day, with a total of 4 times from day 5 to 11 post-immunization. All rats were euthanized on day 13 post-immunization. Bilateral inguinal lymph nodes and spleens were then harvested and grinded through the cell strainer (70 μm) to prepare mononuclear cell (MNC) suspension for further experiments.

### Histology

Histological analysis was performed to evaluate cell infiltration and demyelination. Briefly, sciatic nerves were collected, fixed with 4% paraformaldehyde and embedded in paraffin. Longitudinal sections at thickness of 4 μm were prepared and stained with hematoxylin and eosin (H&E) and Luxol fast blue. Slides were imaged using a Leica DMi8 microscope.

### Co-culture Assays *in vitro*

Splenic MNCs were isolated from healthy rats and seeded into 96-well plates (1 × 106/mL). Cells were activated by α-CD3 (1 μg/mL; G4.18; eBioscience, 16-0030-85) and α-CD28 (1 μg/mL; JJ319; BioGems, 10313-25) for 24 h. In some experiments evaluating the direct effects of exosomes on T cells, splenic T cells were first purified by magnetic separation. Briefly, splenic MNCs were stained with Alexa Fluor 647-conjugated anti-CD3 (Biolegend, 201408) and then incubated with Anti-Cy5/Anti-Alexa Fluor 647 MicroBeads (Miltenyi Biotec, 130-091-395) according to the manufacture's recommendations. Positive selection was performed using an OctoMACS™ Separator (Miltenyi Biotec, 130-042-109). Purified splenic T cells were subsequently seeded into 96-well plates (1 × 10^6^/mL) with plate-bound α-CD3 (1 μg/mL) and α-CD28 (1 μg/mL). After 24 h of activation, splenic MNCs or T cells were co-cultured with exosomes at indicated concentrations for another 48 h and cells were collected for further analysis.

To investigate the effects of macrophage derived exosomes on maturation of dendritic cells. Bone marrow derived dendritic cells (BMDCs) were first prepared as previously described (23). Briefly, bone marrow cells were collected by flushing the femurs and tibias of healthy Lewis rats and grown in RPMI 1640 medium (Biological Industries, 01-100-1A) supplemented with 10% FBS, 1% penicillin-streptomycin, and recombinant rat GM-CSF (10 ng/mL; Biolegend, 592602) + recombinant rat IL-4 (10 ng/mL). Bone marrow cells were cultured in a humidified incubator with 5% CO_2_ at 37°C. Non-adherent cells were discarded after incubation for 3 days and adherent cells were cultured for additional 4 days. On day 7, floating and loosely adherent cells were harvested, induced for maturation by LPS (500 ng/mL) and co-cultured with exosomes for 24 h.

### PKH26 Labeling of Exosomes

Exosomes were fluorescently labeled using PKH26 Red Fluorescent Cell Linker Mini Kit (Sigma-Aldrich, MINI26). Isolated exosomes were first resuspended in Diluent C and stained with PKH26 at room temperature for 5 min. The reaction was then stopped by adding exosome-depleted FBS. Labeled exosomes were washed with PBS, pelleted by ultracentrifugation, and finally resuspended in PBS. To ascertain cellular uptake of exosomes, purified splenic T cells were co-cultured with PKH26-labeled exosomes for 6 h. Samples were washed twice with PBS and imaged using a Leica DMi8 microscope.

### Flow Cytometry

For detection of intracellular cytokines, lymph node and splenic MNCs from EAN rats or cells collected after *in vitro* co-culture assays were incubated within medium containing cell stimulation cocktail plus protein transport inhibitors (eBioscience, 00-4975-03) for 5 h at 37°C, followed by surface staining with fluorescein isothiocyanate (FITC)-conjugated anti-CD4 (Biolegend, 203305) and PE/Cy7-conjugated anti-CD8a (eBioscience, 25-0084-82) for 30 min at 4°C. Cells were then fixed with 2% paraformaldehyde for 20 min at 4°C, permeabilized with permeabilization buffer (eBioscience, 00-8333-56) and stained with PE-conjugated anti-IFN-γ (Biolegend, 507806) and APC-conjugated anti-IL-17A (eBioscience, 17-7177-81) for 30 min at 4°C.

For transcription factor detection, cells were first stained with FITC-conjugated anti-CD4, PE-conjugated anti-CD25 (eBioscience, 12-0390-82), PE/Cy7-conjugated anti-ICOS (Biolegend, 313520) for 30 min 4°C. Cells were then fixed and permeabilized using transcription factor staining buffer set (eBioscience, 00-5523-00) according to the manufacturer's instructions, followed by staining with APC-conjugated anti-Foxp3 (eBioscience, 17-5773-82) for 40 min at room temperature.

Cell proliferation assays were performed as previously described (25). Briefly, splenic MNCs were labeled with carboxyfluorescein diacetate succinimidyl ester (CFSE), and were then cultured under corresponding conditions as described above. Cells were stained with PE-conjugated anti-CD4 (Biolegend, 201507) and PE/Cy7-conjugated anti-CD8a before examination by flow cytometry. In certain experiments, cultured splenic MNCs were first stained with FITC-conjugated anti-CD4 and PE/Cy7-conjugated anti-CD8a, and were then evaluated for cell apoptosis using PE Annexin V Apoptosis Detection Kit with 7-AAD (Biolegend, 640934) according to manufacturer's instructions.

For detection of surface markers on BMDMs, cells were first detached from non-tissue-culture dishes by incubation in ice-cold PBS containing 10 mM EDTA followed by gentle pipetting. Harvested BMDMs were stained with PerCP/Cy5.5 anti-CD11b/c (Biolegend, 201819), combined with rabbit anti-CD163 (Abcam, ab182422) or rabbit anti-CD206 (Abcam, ab64693) for 30 min at 4°C, and stained with Alexa Fluor 488-conjugated goat anti-rabbit IgG (Abcam, ab150077) for 30 min at 4°C.

Other reagents used for surface staining were as follows: PE/Cy7-conjugated anti-CD45R (B220; eBioscience, 25-0460-82), FITC-conjugated Peanut Agglutinin (PNA; Vector labs, FL-1071-10), PE-conjugated anti-CD161a (BD Biosciences, 555009), Alexa Fluor 647-conjugated anti-CD3 (Biolegend, 201408), APC-conjugated anti-CD11b (BD Biosciences, 562102), PE-conjugated anti-CD80 (eBioscience, 12-0800-82), PE-conjugated anti-CD86 (eBioscience, 12-0860-83), FITC-conjugated anti-MHC class II (eBioscience, 11-0920-82).

Stained cells were analyzed using a BD FACSAria II flow cytometer (BD Biosciences), and data were analyzed with FlowJo software.

### Nitric Oxide (NO) Production Detection *in vitro*

Macrophages were polarized to M1 macrophages as described above or left untreated (M0). Culture media were collected 24 and 48 h later, respectively, and examined for the presence and concentration of NO by Griess reagents (Beyotime, S0021).

### ELISA

Conditioned culture media or solutions containing isolated exosomes were detected using ELISA kits for IL-12p70 (Boster, EK1652) or IFN-γ (Dakewe, 1310002) according to the manufacturer's instructions. Briefly, samples were added to microwells pre-coated with specific antibodies to IL-12p70 or IFN-γ. Corresponding biotin-conjugated antibodies were then added and after incubation, unbound antibodies were removed by a wash step. Streptavidin-HRP was subsequent added, followed by a wash step to remove unbound HRP. TMB were added and the reaction was terminated by acid. The absorbance was measured by a microplate reader at 450 nm.

### Statistical Analysis

Data are presented as means ± SEM. Test of Normality and test of homogeneity of variance were performed by Shapiro-Wilk test and Levene's test, respectively. For data with normal distribution, hypothesis testing was performed by Student's *t*-test or one-way ANOVA. Multiple comparisons were conducted by LSD test with equal variances or Dunnett's T3 test without equal variances. For data without normal distribution, non-parametric test (Kruskal-Wallis test) was applied, followed by pairwise comparisons. Statistical analyses were performed with SPSS statistics 22.0 (IBM). Statistically significant difference is indicated as ^*^p < 0.05, ^**^p < 0.01, and ^***^p < 0.001.

## Results

### Establishment of Polarized Bone Marrow Derived Macrophages (BMDMs) and Isolation of Macrophage-Derived Exosomes

**Figure 1:**
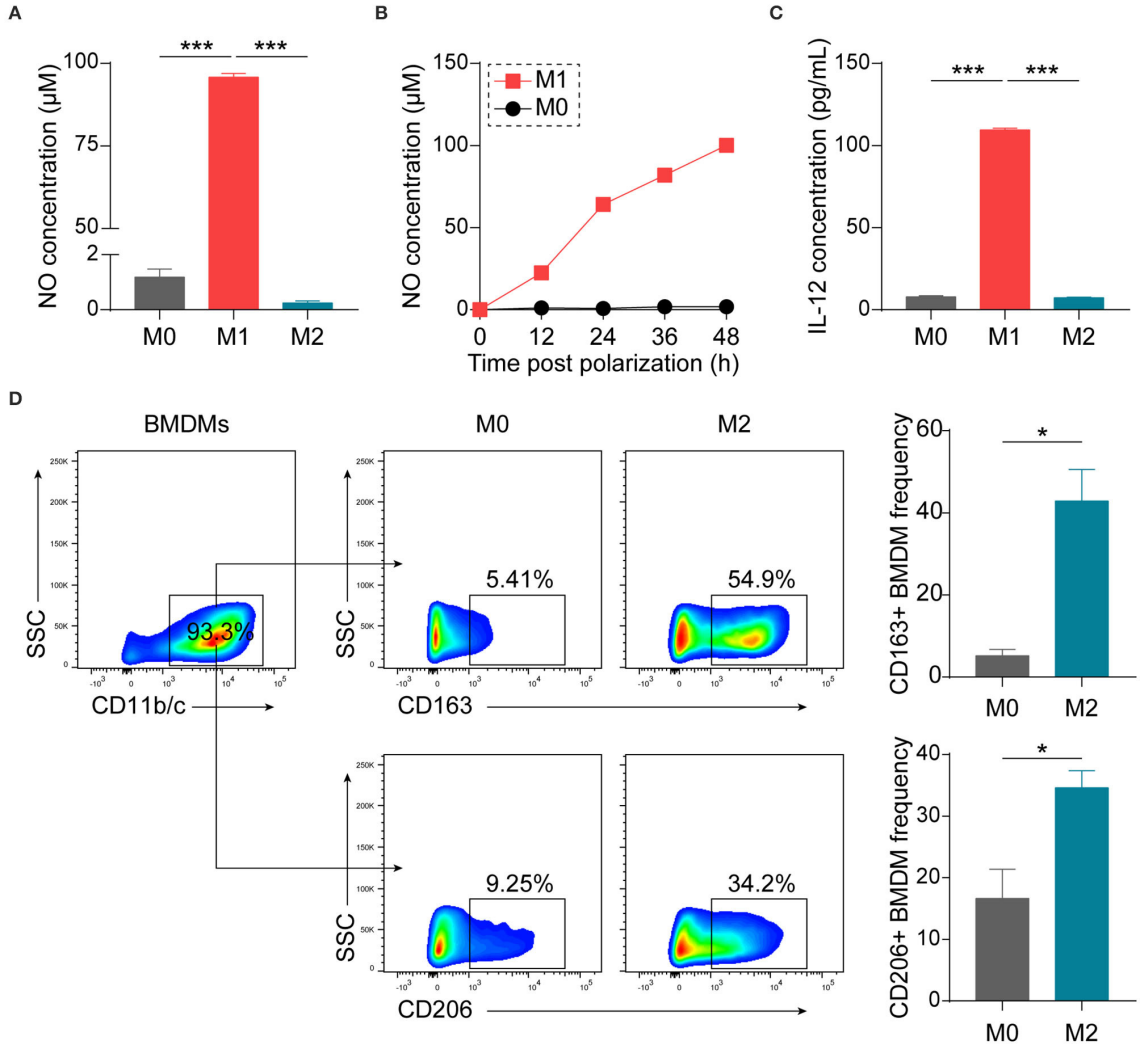
Establishment and characterization of differently polarized macrophages *in vitro*. BMDMs, generated from bone marrow precursor cells with M-CSF, were polarized to M1 macrophages with LPS (100 ng/mL) + IFN-γ (20 ng/mL) or to M2 macrophages with IL-4 (20 ng/mL) at day 7. **(A)** Assessment of NO production 48 h after polarization by Griess assay (*n* = 4 in each group). **(B)** Kinetics of NO production by macrophages (*n* = 3 in each time point). **(C)** Assessment of IL-12p70 production 24 h after polarization by ELISA (*n* = 3 in each group). **(D)** Representative flow cytometry plots and summary data showing M2 macrophages using CD163 or CD206 as the markers (*n* = 4 in each group). Data are expressed as mean ± SEM. *p < 0.05, ***p < 0.001 calculated by Student's *t*-test or one-way ANOVA followed by LSD test with normally distributed data.

Considering the convenience and good reproducibility of BMDMs in studying macrophage biology (26–28), we generated BMDMs from bone marrow precursor cells of Lewis rats with M-CSF. BMDMs were polarized with LPS + IFN-γ or IL-4 to obtain M1 or M2 phenotypes, respectively. Polarized M1 macrophages were confirmed by examining production of NO (Figures 1A,B) and IL-12, one of typical M1 cytokines (Figure 1C). To verify polarized M2 macrophages, we performed flow cytometry and observed increases in the expression of CD163 as well as CD206 (Figure 1D).

**Figure 2:**
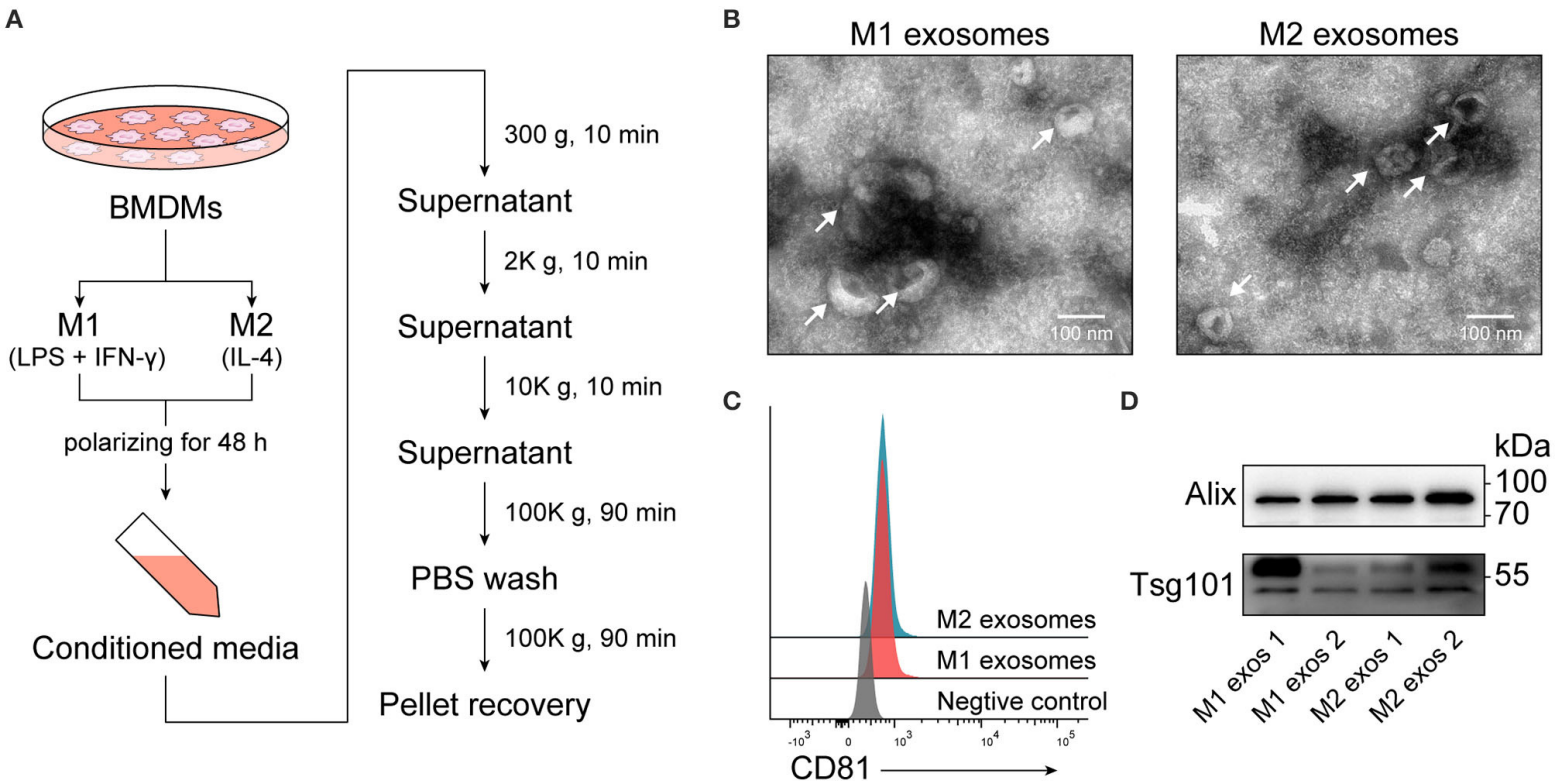
Isolation and characterization of exosomes derived from M1 or M2 macrophages. **(A)** Schematic illustrating duration of polarization and procedure for isolation of exosomes. **(B)** Electron microscopic images providing morphological characteristics of isolated macrophage derived exosomes. **(C)** Representative flow cytometry histograms depicting the expression of CD81, an exosome-enriched surface molecule, on exosomes passively absorbed to aldehyde-sulfate latex beads. **(D)** Immunoblot analysis of TSG101 and ALIX in exosomal proteins.

Conditioned media from M1 or M2 macrophages were collected and exosomes were subsequently isolated by differential centrifugation (Figure 2A), which is a widely used method balancing quantity and purity (29). Recovered exosomes were examined by transmission electron microscopy, and intact exosomes with typical morphology and size range (50–150 nm) could be observed (Figure 2B). Exosomes were then passively absorbed to 4 μm aldehyde-sulfate latex beads, making them detectable by flow cytometers, and the expression of CD81, one of the surface molecules enriched in exosomes, was identified (Figure 2C). The presence of another two exosome-enriched proteins, TSG101, and ALIX, was confirmed by immunoblotting (Figure 2D).

### M1 Exosomes Exacerbate EAN via Boosting Cellular Immunity *in vivo*

**Figure 3:**
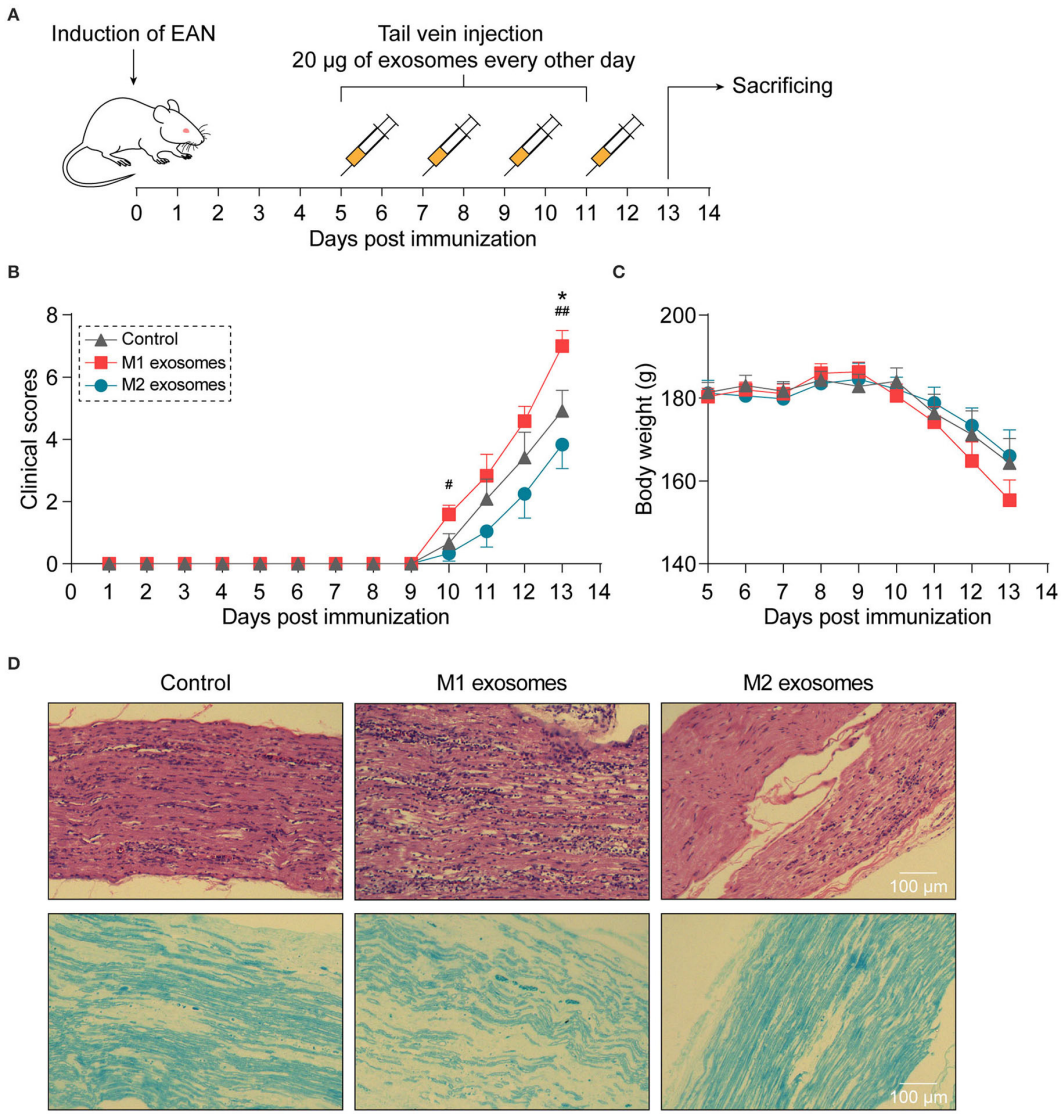
Different effects of M1 or M2 exosomes on the development of EAN. **(A)** Schematic illustration of experimental design for investigating the effects of M1 or M2 exosomes on development of EAN which was established by immunizing Lewis rats with BPM. **(B,C)** Clinical scores **(B)** and body weight **(C)** of EAN rats (*n* = 6 in each group) were evaluated and recorded every day. **(D)** Representative histology of sciatic nerves at day 13 post-immunization. H&E staining (upper), Luxol fast blue staining (lower). Data are expressed as mean ± SEM. *p < 0.05 vs. the control group, ^#^*p* < 0.05, ^##^*p* < 0.01 vs. the group of M2 exosomes, calculated by Kruskal-Wallis test followed by pairwise comparisons with not normally distributed data (day 10) or by one-way ANOVA followed by LSD test with normally distributed data at other time points.

To investigate the effects of M1 and M2 exosomes on the development of EAN, rats were randomly assigned to different groups and given 20 μg of exosomes or equal volume of vehicle via tail vein every other day from day 5 to day 11 post-immunization (Figure 3A). The results showed that M1 exosomes aggravated EAN disease severity, while M2 exosomes exhibited potentials to mitigate the development of EAN (Figures 3B,C). Histological assessments were then conducted to confirm the infiltration of inflammatory cells (Figure 3D, upper panel) and demyelination (Figure 3D, lower panel) in sciatic nerves.

**Figure 4:**
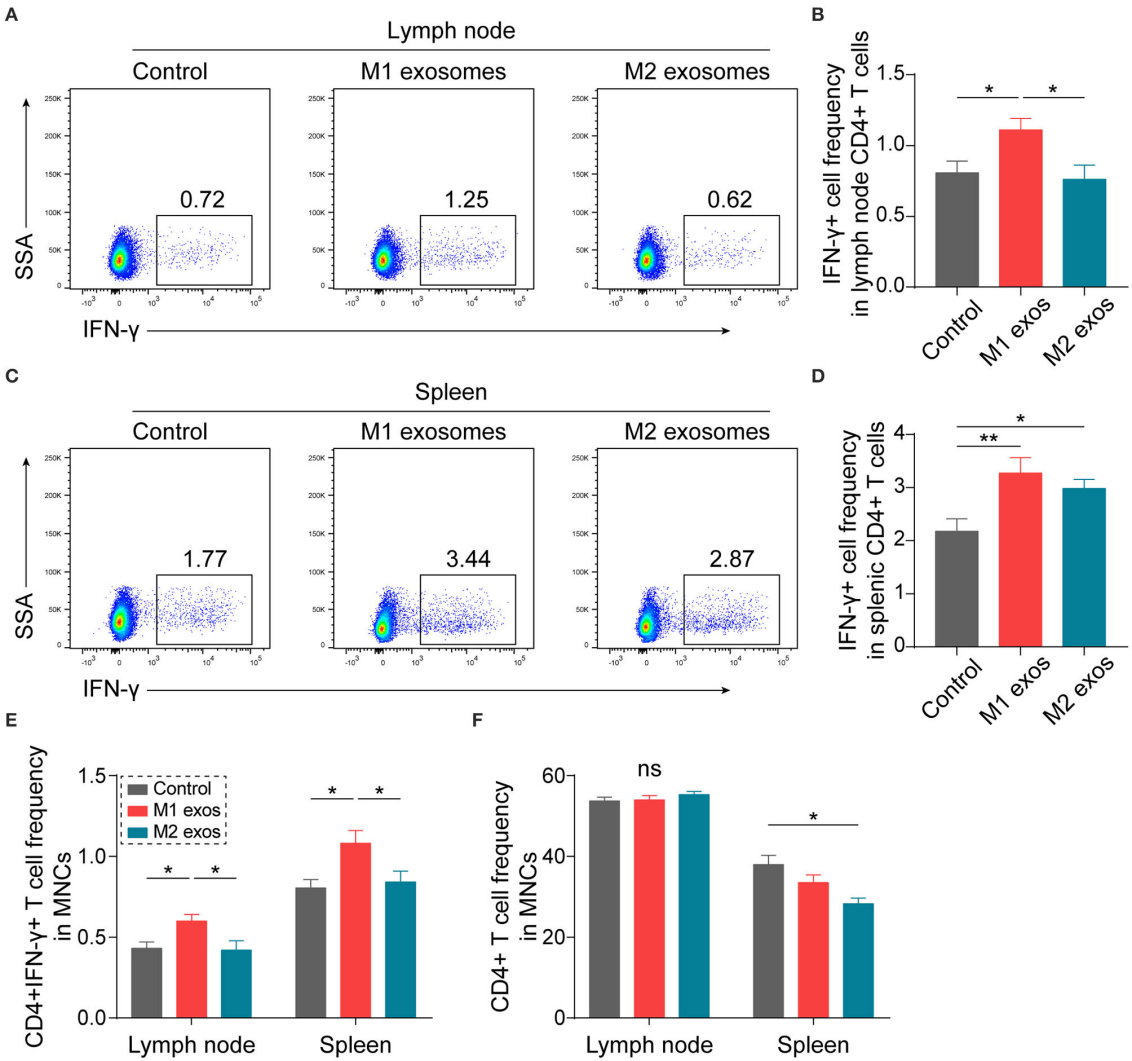
M1 exosomes boost Th1 response while M2 exosomes fail to restrain Th1 cell expansion in EAN. Lymph node and splenic MNCs from EAN rats at the disease progressive stage (day 13) were harvested and evaluated for IFN-γ expression by flow cytometry (*n* = 6 in each group). **(A,B)** Representative flow cytometry plots (**A**, gated on CD4+ cells) and summary data **(B)** illustrating the expression of IFN-γ in lymph node CD4+ T cells. **(C,D)** Representative flow cytometry plots (**C**, gated on CD4+ T cells) and summary data **(D)** illustrating the expression of IFN-γ in splenic CD4+ T cells. **(E)** Summary data illustrating the proportion of CD4+IFN-γ+ T cells in MNCs. **(F)** Summary data illustrating the proportion of CD4+ T cells in MNCs. Data are expressed as mean ± SEM. *p < 0.05, **p < 0.01, ns, not significant, calculated by one-way ANOVA followed by LSD test with normally distributed data **(B,D,E)** or by Kruskal-Wallis test followed by pairwise comparisons with not normally distributed data (**F**, right).

**Figure 5:**
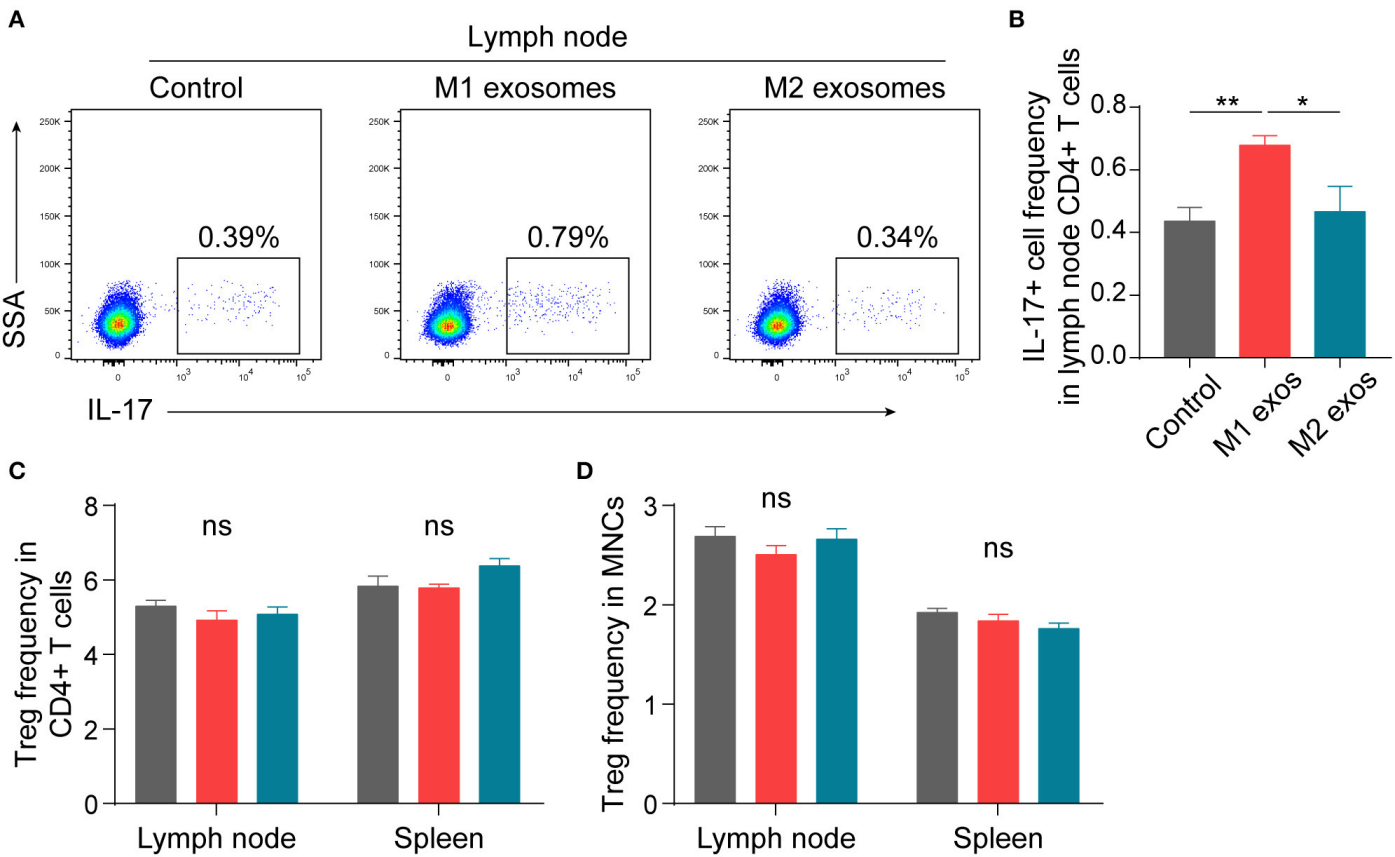
Effects of M1 and M2 exosomes on Th17 or Treg cells in EAN. Th17 and Treg cells were detected in lymph node and splenic MNCs from EAN rats by flow cytometry (*n* = 6 in each group). **(A,B)** Representative flow cytometry plots (**A**, gated on CD4+ T cells) and summary data **(B)** illustrating the expression of IL-17 in lymph node CD4+ T cells. **(C,D)** Summary data illustrating the proportion of Treg (CD25+Foxp3+) cells in CD4+ T cells **(C)** and the proportion of Treg (CD4+CD25+Foxp3+) cells in MNCs **(D)**. Data are expressed as mean ± SEM. *p < 0.05, **p < 0.01, ns, not significant, calculated by one-way ANOVA followed by LSD test with normally distributed data **(B–D)**.

In order to ascertain the immunological basis behind the observed effects, EAN rats were sacrificed at day 13 post-immunization, and splenic MNCs were isolated and examined by flow cytometry. Intriguingly, we found that M1 exosomes increased the proportions of IFN-γ producing CD4+ T cells from both draining lymph nodes and spleens (Figures 4A–D). Besides, an unexpected increase in IFN-γ producing splenic CD4+ T cells was also observed following M2 exosome treatment (Figures 4C,D). Further analysis showed that the proportions of IFN-γ+CD4+ T cells in total MNCs were increased by M1 exosomes but not by M2 exosomes (Figure 4E), in that M2 exosomes caused a significant decrease in splenic CD4+ T cells (Figure 4F). These results suggest that M1 exosomes significantly promote Th1 response in EAN and M2 exosomes fail to inhibit Th1 cell expansion. Nevertheless, there was no change in CD8+ T cell frequency and IFN-γ expression in CD8+ T cells (data not shown). In addition, M1 exosomes increased IL-17 expressing CD4+ T cells from draining lymph nodes, indicating an increased Th17 response, while M2 exosomes were not able to inhibit Th17 response (Figures 5A,B). We also found that the effects of M1 exosomes were not a consequence accompanying changes in regulatory T (Treg) cells (Figures 5C,D).

In summary, these results suggest that M1 exosomes promote EAN progression via boosting Th1 and Th17 response, and M2 exosome treatment causes a trend toward alleviated disease severity, possibly via a mechanism not involving regulation of Th1 and Th17 cells.

### Effects of Macrophage-Derived Exosomes on Germinal Center Reactions and Innate Immunity in EAN

**Figure 6:**
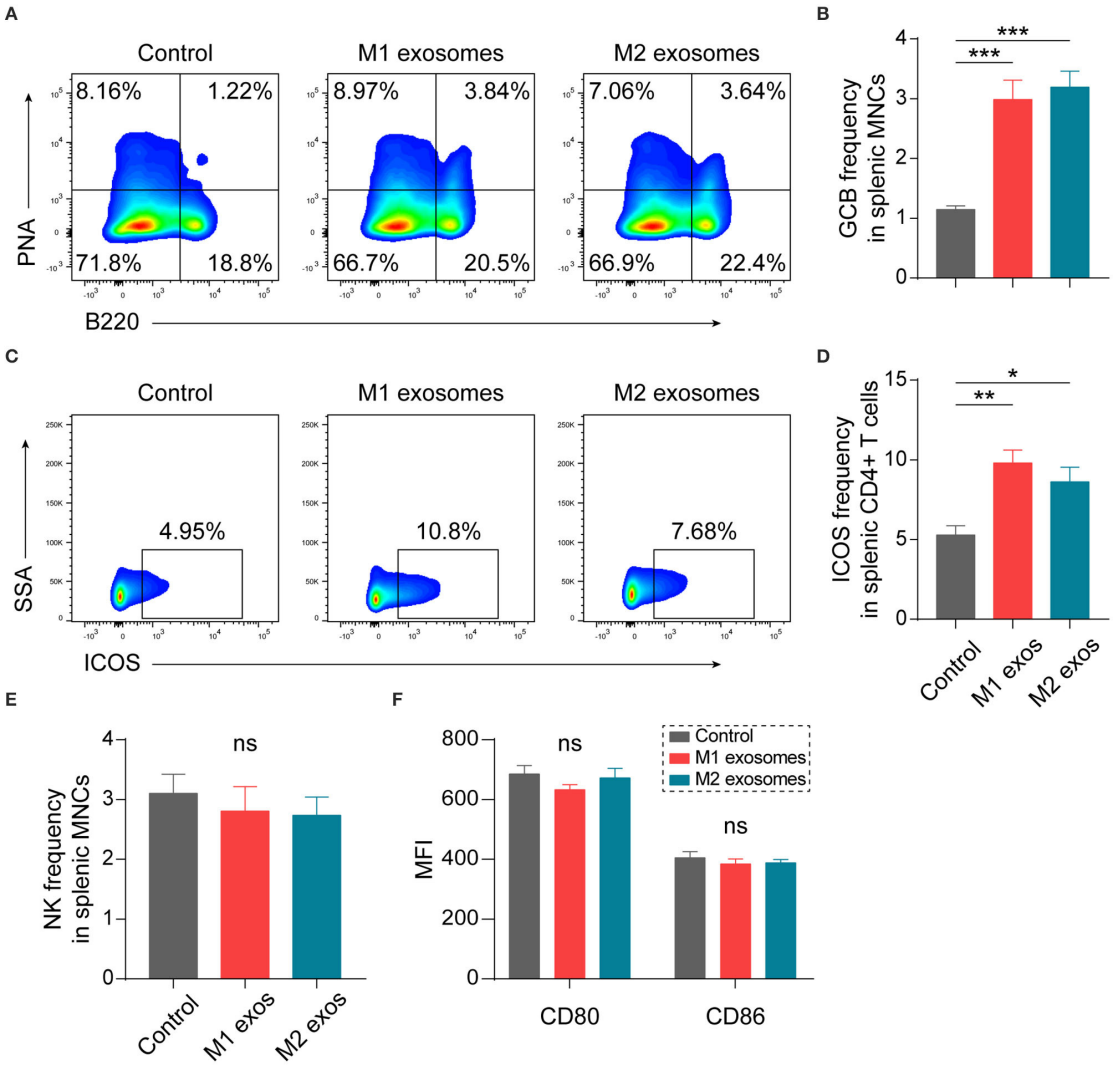
Effects of M1 and M2 exosomes on germinal center reaction and innate immunity. Splenic MNCs from EAN rats were isolated and certain aspects of adaptive and innate immunity were detected by flow cytometry (*n* = 6 in each group). **(A,B)** Representative flow cytometry plots (**A**, gated on splenic MNCs) and summary data **(B)** illustrating the proportion of GCB cells (B220+PNA+) in splenic MNCs. **(C,D)** Representative flow cytometry plots (**C**, gated on CD4+ T cells) and summary data **(D)** illustrating the expression of ICOS in splenic CD4+ T cells. **(E)** Summary data illustrating the proportion of NK cells (CD3-CD161+) in splenic MNCs. **(F)** Summary data illustrating the expression of CD80 and CD86 on splenic macrophages (CD11b+). Data are expressed as mean ± SEM. *p < 0.05, **p < 0.01, ***p < 0.001, ns, not significant, calculated by calculated by one-way ANOVA followed by LSD test with normally distributed data **(B,E,F)** or by Kruskal-Wallis test followed by pairwise comparisons with not normally distributed data **(D)**.

Our previous studies have shown that humoral immunity participates in the development of EAN (24, 30). We found unexpectedly that both M1 and M2 exosomes caused an increase in splenic germinal center B (GCB) cells (Figures 6A,B). Further analysis showed that the expression of ICOS was increased in CD4+ T cells (Figures 6C,D). ICOS plays essential roles in differentiation and maintenance of follicular helper T (Tfh) cells, and subsequent formation and regulation of germinal center reactions (31–33). Thus, these results suggest that M1 and M2 exosomes modulate humoral immunity possibly via upregulation of ICOS on CD4+ T cells.

Certain aspects of innate immunity were also explored by flow cytometry. Treatment with M1 or M2 exosomes did not affect frequency of NK cells (Figure 6E) and expression of CD80 and CD86 on macrophages (Figure 6F) in spleen of EAN rats at least under current treatment scheme.

### M1 Exosomes and M2 Exosomes Differ in Their Capacities To Regulate IFN-γ Expression in T Cells *in vitro*

**Figure 7:**
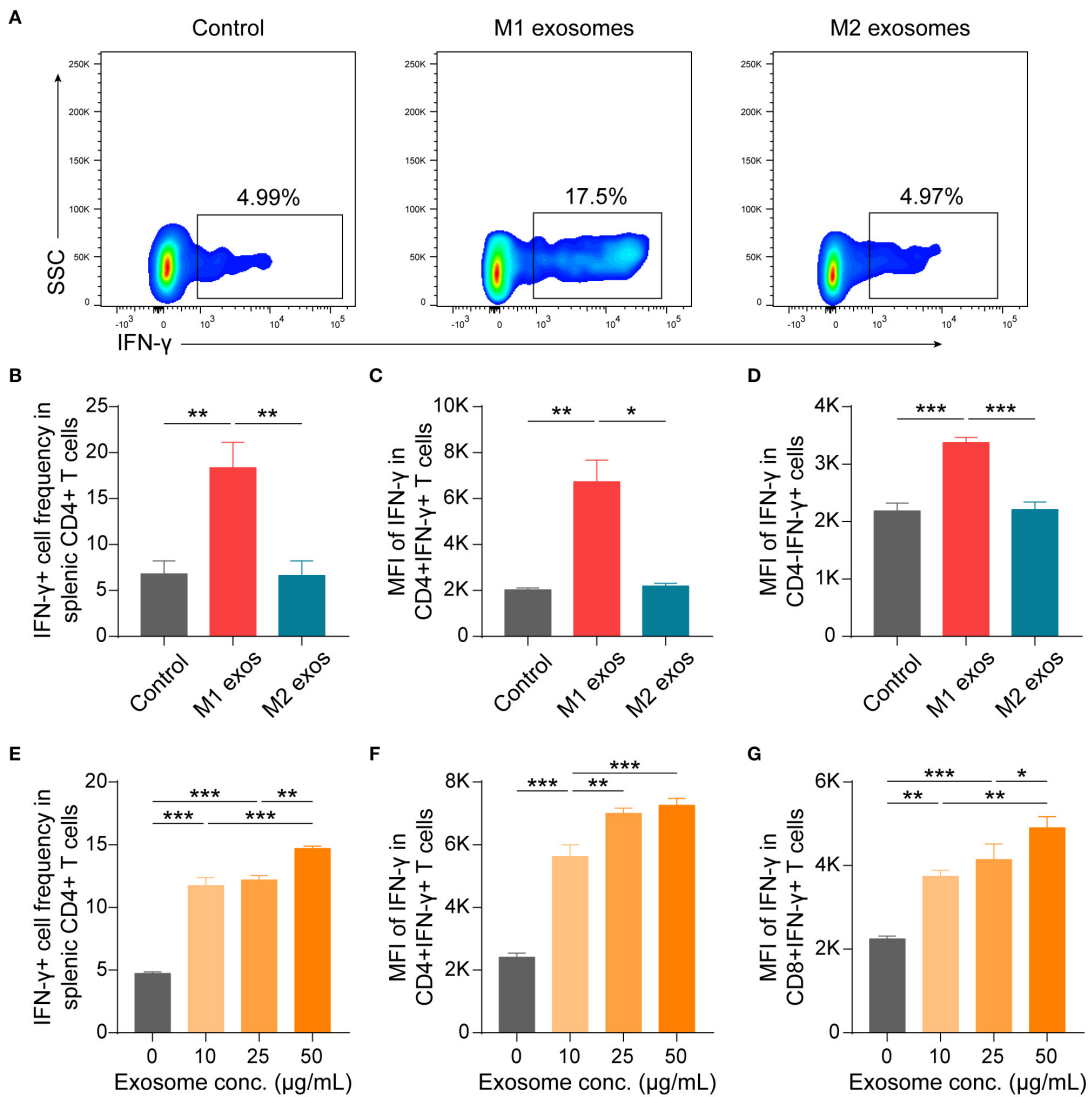
M1 exosomes promote IFN-γ expression in both CD4+ and CD8+ T cells, while M2 exosomes are not capable of inhibiting IFN-γ expression *in vitro*. Splenic MNCs were isolated and co-cultured with M1 or M2 exosomes for 48 h following pre-activation by α-CD3 (1 μg/mL) and α-CD28 (1 μg/mL) for 24 h, and were then detected for IFN-γ expression in CD4+ or CD8+ T cells by flow cytometry. **(A,B)** Representative flow cytometry plots (**A**, gated on CD4+ T cells) and summary data (**B**, *n* = 6 in each group) illustrating the proportion of IFN-γ+ cells in splenic CD4+ T cells after incubating splenic MNCs with macrophage exosomes (50 μg/mL). **(C,D)** Summary data (*n* = 6 in each group) illustrating the MFI of IFN-γ expression in CD4+ cells **(C)** and CD4- cells (D). **(E–G)** Summary data (*n* = 4 in each group) illustrating the proportion of IFN-γ+ cells in CD4+ T cells **(E)**, and MFI of IFN-γ expression in CD4+ T cells **(F)** and in CD8+ T cells **(G)** after incubating splenic MNCs with M1 exosomes at indicated concentrations. Data are expressed as mean ± SEM. *p < 0.05, **p < 0.01, ***p < 0.001, calculated by one-way ANOVA followed by LSD test with normally distributed data **(B,D–G)** or by Kruskal-Wallis test followed by pairwise comparisons with not normally distributed data **(C)**.

To further validate the effects of macrophage derived exosomes on Th1 response, splenic MNCs were incubated with M1 or M2 exosomes *in vitro* and then examined by flow cytometry. We found that M1 exosomes increased both the proportion and intensity of IFN-γ expression in CD4+ T cells, while M2 exosomes exert no significant functions under current conditions (Figures 7A–C). And the promotion effects on IFN-γ by M1 exosomes were not restricted to CD4+ T cells (Figure 7D). The effects of M1 exosomes were further evidenced by demonstrating the existence of dose-effect relationship between concentrations of M1 exosomes and IFN-γ expression in CD4+ T cells (Figures 7E,F). As expected, M1 exosomes increased the proportions (data not shown) and intensities (Figure 7G) of IFN-γ expression in CD8+ T cells as well. We proceeded to examine the levels of IFN-γ in the supernatants using ELISA and confirmed the observations from intracellular cytokine staining (Supplementary Figure 1A). We also detected the concentration of IL-12 and IFN-γ by ELISA in exosome solutions corresponding to the largest quantity used in co-culture assays, and the concentrations were below 2 and 10 pg/mL, respectively (data not shown). Thus, it's reasonable to exclude the confounding effects of exosome-containing or possible isolation-related contaminating cytokines. In addition, we found that M1 exosomes, but not M2 exosomes, were able to promote the proliferation of both CD4+ and CD8+ T cells (Supplementary Figure 1B). Both M1 and M2 exosomes did not affect the apoptosis of T cells under current experimental conditions (Supplementary Figure 1C).

Taken together, these results suggest that M1 exosomes promote both the differentiation and effector function of IFN-γ producing CD4+ and CD8+ T cells, while M2 exosomes are incapable of restricting IFN-γ expression *in vitro*.

### M1 Exosomes Exert Direct Regulatory Functions on T Cells To Promote IFN-γ Production *in vitro*

**Figure 8:**
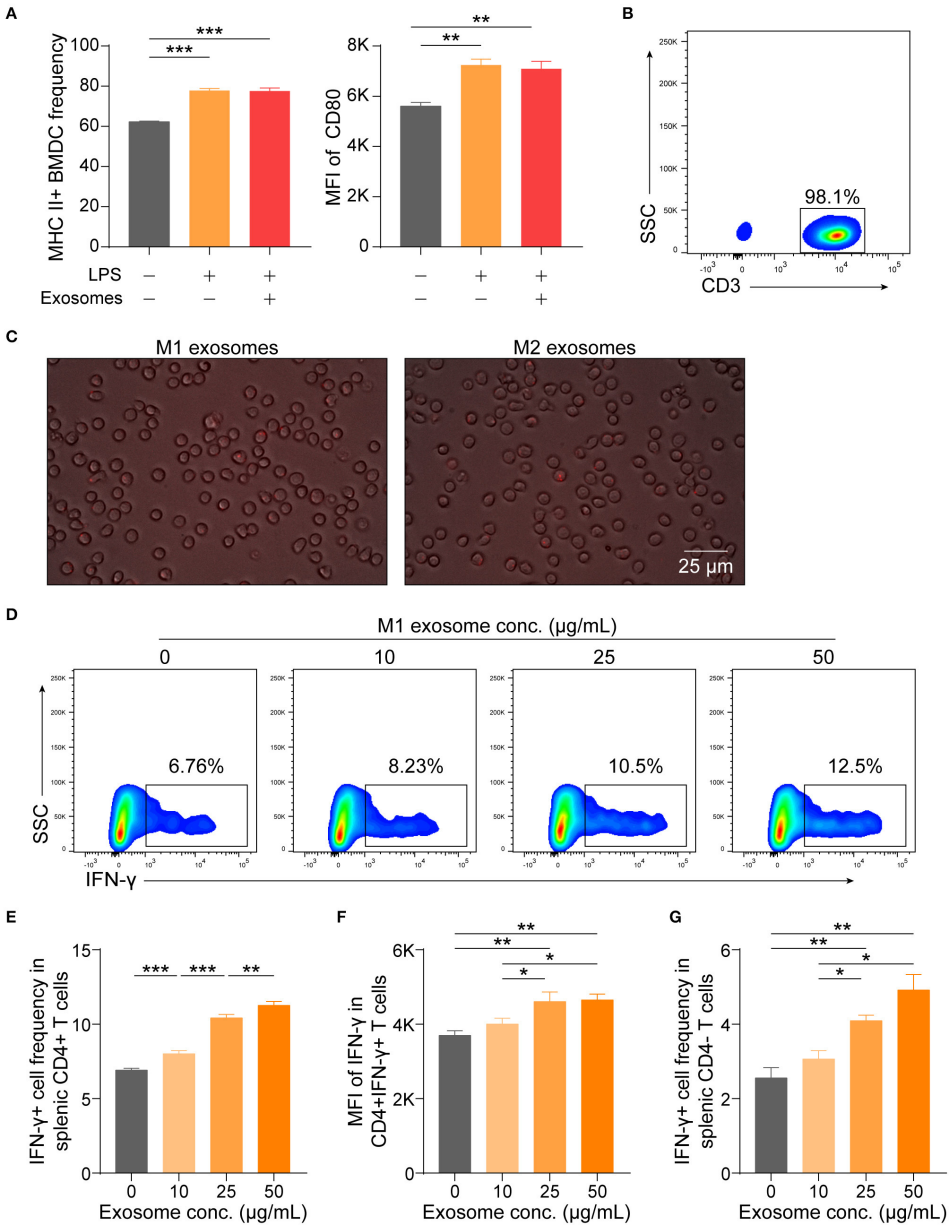
M1 exosomes augment IFN-γ expression in T cells in a direct manner. **(A)** BMDCs, generated from bone marrow precursor cells with GM-CSF + IL-4, were co-cultured with M1 exosomes (50 μg/mL) for 24 h to evaluate the effects of exosomes on BMDC maturation, and expression of MHC II (left) and CD80 (right, gated on MHC II+ cells) were detected by flow cytometry (*n* = 3 in each group). **(B)** Representative flow cytometry plots depicting purity of splenic T cells isolated by magnetic separation. **(C)** Images displaying direct uptake of PKH26-labeled M1 and M2 exosomes by T cells. **(D–G)** Isolated splenic T cells were co-cultured with M1 exosomes at indicated concentrations for 48 h following pre-activation by plate-bound α-CD3 (1 μg/mL) and α-CD28 (1 μg/mL) for 24 h, and were then detected for IFN-γ expression in T cells by flow cytometry (*n* = 6 in each group). Representative flow cytometry plots (**D**, gated on CD4+ cells) and summary data illustrating the proportion of IFN-γ+ cells in CD4+ T cells **(E)**, MFI of IFN-γ expression in CD4+ T cells **(F)**, and the proportion of IFN-γ+ cells in CD4- T cells **(G)**. Data are expressed as mean ± SEM. *p < 0.05, **p < 0.01, ***p < 0.001, calculated by one-way ANOVA, with normally distributed data, followed by LSD test with equal variances **(A,E,F)** or Dunnett's T3 test without equal variances **(G)**.

Although M1 macrophages have been proven capable of amplifying Th1 response, knowledge about the underlying mechanisms has long been confined to functions of various cytokines, such as IL-12 and IL-18 (8, 9, 34, 35). These cytokines are also of importance for effector function of CD8+ T cells (36). Thus, it was intriguing for us to find that M1 exosomes could function in promoting IFN-γ expression in T cells both *in vivo* and *in vitro* and we then wondered whether the observed phenotypes were due to direct effects on T cells. Therefore, we first evaluated the effects of M1 exosomes on maturation of BMDCs, a process pivotal for T cell activation and differentiation, and found that M1 exosomes did not further upregulate expression of MHC II and CD80 (Figure 8A). Splenic T cells were then purified by magnetic separation (Figure 8B), and co-cultured with PKH26-labeled M1 and M2 exosomes, respectively. After 6 h, T cells exhibited uptake of both M1 and M2 exosomes, as indicated by the presence of red fluorescence within cells (Figure 8C). We proceeded to examine the direct effects of M1 exosomes on expression of IFN-γ in T cells by flow cytometry analysis. Dose-dependent increases in the proportions (Figures 8D,E) and intensities (Figure 8F) of IFN-γ expression in CD4+ T cells were observed. There also existed dose-dependent increases in the proportions (Figure 8G) and intensities (data not shown, not statistically significant due to insufficient power) of IFN-γ expression in CD4- T cells.

These results, combined with preceding results, indicate that M1 exosomes exert direct effects on T cells to promote the differentiation and effector function of IFN-γ producing CD4+ and CD8+ T cells *in vitro*.

## Discussion

In this study, we evaluated the effects of macrophage-derived exosomes on the development of EAN and explored the underlying immunological basis. Our *in vivo* and *in vitro* investigations about the effects of M1 exosomes on IFN-γ expression in T cells provided evidence that M1 exosomes aggravated EAN, at least in part, via directly modulating the differentiation and function of Th1 effector cells. Whereas, M2 exosomes were not able to restrain Th1 response but still showed potential to attenuate EAN. Our findings reveal novel mechanisms by which macrophages participate in the development of EAN and extend our understanding about the crosstalk network between macrophages and T cells.

Emerging studies support the involvement of macrophages in the development of neuroinflammation affecting peripheral nervous system or central nervous system, and certain cytokines secreted by macrophages have been identified to play indispensable roles in these processes (10, 13, 37–40). After polarization to M1 phenotypes by combination of IFN-γ from Th1 cells and other environmental changes, macrophages synthesize and release IL-12, which has been considered to be one of the most important environmental clues for Th1 lineage commitment, thus forming a positive feedback and amplifying type 1 immunity (34, 41, 42). In this study, we found that M1 exosomes could deteriorate EAN, accompanied by enhanced Th1 response *in vivo*. We then performed *in vitro* assays and our results suggest, for the first time, that M1 exosomes could exert direct regulatory effects on T cells, promoting both differentiation (increased proportions) and effector function (increased IFN-γ intensities) of Th1 cells, and increasing IFN-γ production by CD8+ T cells as well. Considering that M1 exosomes have also been demonstrated to induce expression of pro-inflammatory cytokines, such as IL6 and IL-12, in dendritic cells and macrophages (43), it is possible that M1 exosomes are also able to promote Th1 and Th17 response in an indirect manner. Thus it is reasonable to speculate that the amplification of cellular immunity response in EAN *in vivo* by M1 exosomes might be attributed to integration of parallel direct and indirect effects.

The molecular basis underlying the effects of M1 exosomes on T cells would be worthy of further explorations. T-bet, a member of the T-box family of transcription factors, has been accepted as the master regulator of Th1 cells, promoting the differentiation and effector functions of Th1 cells (44, 45), and also plays important roles in sustaining functions of effector CD8+ T cells (46, 47). Combined with our findings on the effects of M1 exosomes on IFN-γ production in both CD4+ T cells and CD8+ T cells *in vivo* or *in vitro*, we are inspired to assume that M1 exosomes might exert effects on T cells via regulation of T-bet, which need ascertaining by further studies. In addition to the effect on IFN-γ production, we found that M1 exosomes promoted the proliferation of CD4+ and CD8+ T cells. Metabolic reprogramming is emerging as a fundamental regulator of T cell activation, differentiation and effector function, and certain biological processes of T cells engage both common and distinct metabolic pathways, in which aerobic glycolysis can support both proliferation and IFN-γ production of activated T cells (48–52). It is worth mentioning that exosomes derived from TAMs enhance aerobic glycolysis of breast cancer cells (19). Therefore, the possibility that M1 exosomes regulate Th1 cells and CD8+ T cells via facilitating metabolic reprogramming also merit consideration.

In addition to the well-characterized roles in initiation and development of inflammation, macrophages have recently been shown to restrict inflammation and facilitate resolution of neuroinflammation and inflammation involving other systems, via upregulation of PD-L1, secretion of anti-inflammatory cytokines as well as pro-resolving lipid mediators, and clearance of apoptotic cells, a process termed efferocytosis (15, 53–55). Our results provide a clue to the therapeutic potentials of exosomes derived from anti-inflammatory macrophages for EAN/GBS. Although causing a significant decrease in splenic CD4+ T cell frequency in EAN, M2 exosomes failed to restrict the overall magnitude of Th1 and Th17 response in the current study, suggesting a mechanism bypassing these effector T cell responses. Indeed, M2 exosomes have been shown to induce the expression of IL-4 and IL-10 in dendritic cells and macrophages and possess the capacity to reprogram M1 macrophages to anti-inflammatory phenotypes *in vitro* (43, 56), rationalizing the attenuating effects of M2 exosomes on EAN. The statistically non-significant result (Figure 3B) might result from relative high standard deviation and inadequate sample size. In addition, compared to the study demonstrating that M2 exosomes expedite wound healing *in vivo* (56), we used a relative small amount of exosomes for assessment of the effects of M2 exosomes on EAN(a total of 80 μg for rats in our study vs. a total of 200 μg for mice). There also exists the possibility that M2 exosomes play a more important role in the resolution phase of neuroinflammation. Altogether, further studies with adjusted treatment scheme are warranted to investigate the therapeutic effects of M2 exosomes on EAN/GBS and other inflammatory disorders.

In conclusion, our results suggest that M1 and M2 exosomes differ in their effects on the development of EAN and that M1 macrophages are able to directly regulate Th1 cells and CD8+ T cells via exosomes, hinting at the intriguing idea that exosomes serve as a bridge between innate and adaptive immunity. A more comprehensive understanding of the communication between macrophages and other immune cells and of the ways by which macrophages participate in the development and resolution of inflammatory disorders would greatly advance the development of targeted therapy.

## Data Availability Statement

The datasets generated for this study are available on request to the corresponding author.

## Ethics Statement

The animal study was reviewed and approved by the Institutional Animal Care and Use Committee at Shandong University School of Medicine.

## Author Contributions

R-SD, BY, and Y-CD designed and supervised the research. TD, C-LY, M-RG, PZ, HL, and X-LL executed the *in vivo* experiments and collected data. TD, YL, and TL performed the *in vitro* experiments and collected data. TD, C-LY, and Y-DL analyzed the data. R-SD and TD wrote the manuscript. All authors have read and approved the final manuscript.

## Conflict of Interest

The authors declare that the research was conducted in the absence of any commercial or financial relationships that could be construed as a potential conflict of interest.
