# Supplementary material for: M1 Macrophage Derived Exosomes Aggravate Experimental Autoimmune Neuritis via Modulating Th1 Response
Source: Front Immunol. 2020 Jul 23;11:1603. doi: 10.3389/fimmu.2020.01603 (PMC7390899; doi:10.3389/fimmu.2020.01603)
Supplement: Supplementary file 1 [file Data_Sheet_1.PDF]

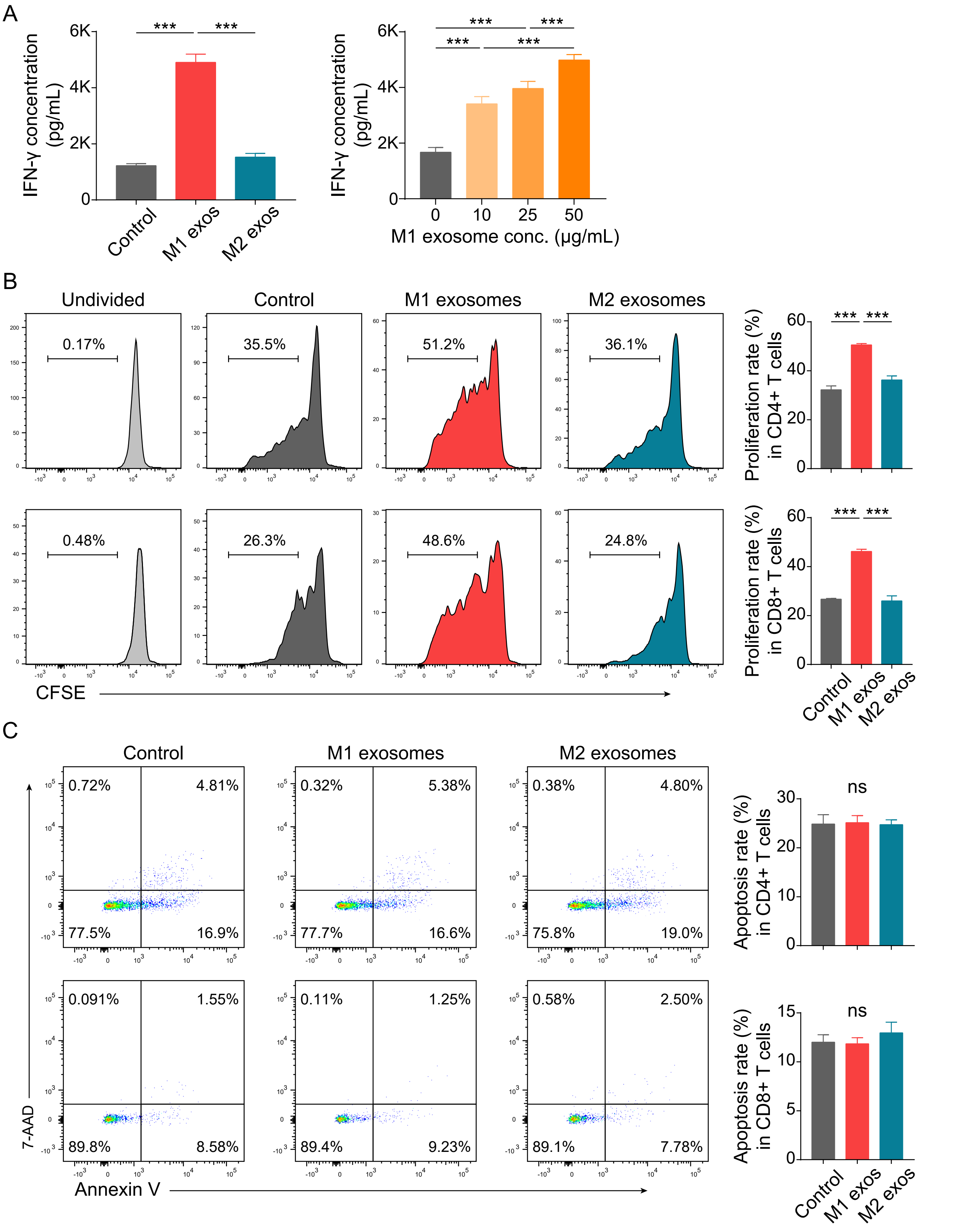

Supplementary Figure 1. M1 and M2 exosomes differ in their capacities to regulate IFN-γ production and proliferation of T cells *in vitro*

Splenic MNCs were isolated and co-cultured with M1 or M2 exosomes for 48 h following pre-activation by α-CD3 (1 µg/mL) and α-CD28 (1 µg/mL) for 24 h. IFN-γ in supernatants were detected by ELISA; proliferation and apoptosis of T cells were examined by flow cytometry. **(A)** IFN-γ concentration in supernatants (n = 4 in each group). **(B)** Representative flow cytometry plots and summary data (n = 4 in each group) illustrating the proliferation of CD4+ T cells (upper panel) and CD8+ T (lower panel) cells after incubating splenic MNCs with macrophage exosomes (50 µg/mL). **(C)** Representative flow cytometry plots and summary data (n = 4 in each group) illustrating the apoptosis of CD4+ T cells (upper panel) and CD8+ T (lower panel) cells. Data are expressed as mean ± SEM. *p < 0.05, **p < 0.01, ***p < 0.001, calculated by one-way ANOVA followed by LSD test with normally distributed data (A, B, C lower) or by Kruskal-Wallis test with not normally distributed data (C upper).
