# Supplementary material for: M1 Macrophage Derived Exosomes Aggravate Experimental Autoimmune Neuritis via Modulating Th1 Response
Source: Front Immunol. 2020 Jul 23;11:1603. doi: 10.3389/fimmu.2020.01603 (PMC7390899; doi:10.3389/fimmu.2020.01603)
Supplement: Supplementary file 2 [file Data_Sheet_2.PDF]

Supplementary Table 1

Reagent	Source	Identifier
**Flow Cytometry**		
anti-rat CD11b (WT.5) APC	BD biosciences	Cat# 562102
anti-rat CD11b/c (OX42) Percp-Cy5.5	Biolegend	Cat# 201819
anti-rat CD161a (10/78) PE	BD biosciences	Cat# 555009
anti-rat CD163	Abcam	Cat# ab182422
anti-rat CD206	Abcam	Cat# ab64693
anti-rat CD25 (OX39) PE	eBioscience	Cat# 12-0390-82
anti-rat CD3 (IF4) Alexa Fluor 647	Biolegend	Cat# 201408
anti-rat CD4 (OX35) FITC	Biolegend	Cat# 203305
anti-rat CD4 (W3/25) PE	Biolegend	Cat# 201507
anti-rat CD45R (HIS24) PE-Cy7	eBioscience	Cat# 25-0460-82
anti-rat CD80 (3H5) PE	eBioscience	Cat# 12-0800-82
anti-rat CD81 (Eat-2) APC	Biolegend	Cat# 104909
anti-rat CD86 (24F) PE	eBioscience	Cat# 12-0860-83
anti-rat CD8a (OX8) PE-Cy7	eBioscience	Cat# 25-0084-82
anti-rat Foxp3 (FJK-16s) APC	eBioscience	Cat# 17-5773-82
anti-rat ICOS (C398.4A) PE-Cy7	Biolegend	Cat# 313520
anti-rat IFN-γ (DB-1) PE	Biolegend	Cat# 507806
anti-rat IL-17A (eBio17B7) APC	eBioscience	Cat# 17-7177-81
anti-rat MHC class II (HIS19) FITC	eBioscience	Cat# 11-0920-82
Peanut Agglutinin FITC	Vector labs	Cat# FL-1071-10
**Immunoblotting**		
anti-ALIX	Abcam	Cat# ab186429
anti-TSG101	Proteintech	Cat# 14497-1-AP
***in vitro* T cell activation**		
anti-rat CD3 (eBioG4.18)	eBioscience	Cat# 16-0030-85
anti-rat CD28 (JJ319)	BioGems	Cat# 10313-25
